# Retraction: Metformin inhibits the proliferation of benign prostatic epithelial cells

**DOI:** 10.1371/journal.pone.0312101

**Published:** 2024-10-09

**Authors:** The PLOS ONE Editors

Following the publication of this article [1] and correction [2], additional concerns were raised regarding the underlying data. Upon editorial follow-up, the corresponding author provided the available underlying data for all figures. Editorial assessment identified discrepancies in the underlying data for Figs 3 and 4, including:

- The original uncropped western blot images provided for multiple panels do not match the published images;
- The experimental conditions described in some of the original data do not match the conditions described in multiple published panels.

The corresponding author (ZW) stated that the unmatching original western blot images are likely from replicate experiments. In relation to the inconsistencies in labels between the original and published results, the corresponding author stated that the original data were mislabeled.

The *PLOS ONE* Editors retract this article due to inconsistencies in data reporting that raise concerns about the reliability of the reported results.

ZW and RG agreed with the retraction. XX, JL, CWJ, CR, and AFO either did not respond directly or could not be reached. ZW stands by the article’s findings and apologizes for the issues with the published article.
